# Genomic Context Analysis of *de Novo STXBP1* Mutations Identifies Evidence of Splice Site DNA-Motif Associated Hotspots

**DOI:** 10.1534/g3.118.200080

**Published:** 2018-02-22

**Authors:** Mohammed Uddin, Marc Woodbury-Smith, Ada J. S. Chan, Ammar Albanna, Berge Minassian, Cyrus Boelman, Stephen W. Scherer

**Affiliations:** * Mohammed Bin Rashid University of Medicine and Health Sciences, College of Medicine, 505055 Dubai, United Arab Emirates 505055,; † The Centre for Applied Genomics,; § Program in Genetics and Genome Biology,; §§ Division of Neurology, and; *** McLaughlin Centre, University of Toronto, The Hospital for Sick Children, Toronto, Ontario, Canada M5G 1X8,; ‡ Institute of Neuroscience NE2 4HH is the postal code, Newcastle University, Newcastle upon Tyne, United Kingdom,; ** Department of Molecular Genetics and; †† Division of Neurology, BC Children’s Hospital, Vancouver, BC, Canada V6H 3N1, and; ‡‡ Al Jalila Children’s Specialty Hospital, Dubai, United Arab Emirates PO Box: 76662

**Keywords:** genome context, epilepsy encephalopathy, loss of function mutation, DNA motif, mutation etiology, Mutant Screen Report

## Abstract

Mutations within *STXBP1* have been associated with a range of neurodevelopmental disorders implicating the pleotropic impact of this gene. Although the frequency of *de novo* mutations within *STXBP1* for selective cohorts with early onset epileptic encephalopathy is more than 1%, there is no evidence for a hotspot within the gene. In this study, we analyzed the genomic context of *de novo STXBP1* mutations to examine whether certain motifs indicated a greater risk of mutation. Through a comprehensive context analysis of 136 *de novo*/rare mutation (SNV/Indels) sites in this gene, strikingly 26.92% of all SNV mutations occurred within 5bp upstream or downstream of a ‘GTA’ motif (*P* < 0.0005). This implies a genomic context modulated mutagenesis. Moreover, 51.85% (14 out of 27) of the ‘GTA’ mutations are splicing compared to 14.70% (20 out of 136) of all reported mutations within *STXBP1*. We also noted that 11 of these 14 ‘GTA’ associated mutations are *de novo* in origin. Our analysis provides strong evidence of DNA motif modulated mutagenesis for *STXBP1 de novo* splicing mutations.

Heterozygous mutations in the brain expressed gene *STXBP1* (MIM #602926) are highly penetrant for neurodevelopmental phenotypes, with the most striking association with early onset epilepsy. This commonly presents as early infantile epileptic encephalopathy (EIEE, also known as Ohtahara Syndrome), and often evolves to severe progressive epileptic disorders such as West Syndrome and Lennox-Gastaut Syndrome. Other frequently described *STXBP1*-associated neurodevelopmental phenotypes include intellectual disability (ID), Autism Spectrum Disorder (ASD), other epilepsy syndromes such as atypical Dravet syndrome, and a variety of movement disorders (Barcia *et al.* 2013; Barcia *et al.* 2014; Boutry-Kryza *et al.* 2015; Carvill *et al.* 2014; Di Meglio *et al.* 2015; Gburek-Augustat *et al.* 2016; Hamdan *et al.* 2009; Lopes *et al.* 2016; Mignot *et al.* 2011; Saitsu *et al.* 2011; Yuen *et al.* 2016; Stamberger *et al.* 2016). *STXBP1* is expressed specifically in brain (Uddin *et al.* 2014) and is involved in the synaptic release of neurotransmitters, with heterozygous mutations resulting in a reduction of both *STXBP1*’s protein product and the functionally related syntaxin-1, both crucial to the presynaptic machinery (Patzke *et al.* 2015; Yamashita *et al.* 2016). Its highly penetrant association with neurodevelopmental phenotypes is therefore unsurprising, indicating this as an important brain development gene.

Mutations in *STXBP1* described in the literature so far comprise more than 136 different single nucleotide variants (SNVs) and small insertion/deletion (indel) seemingly randomly spread throughout the gene: visible clustering is not observed at either the nucleotide or the protein domain levels. A similar pattern of dense mutations is seen in other genes, such as *CFTR* (De Boeck *et al.* 2014) (MIM #602421) and *MECP2 (*Leonard *et al.* 2017) (MIM #300005), both of which comprise mutation spectra occurring across each gene. The mechanisms whereby these mutations occur are largely poorly understood, although a growing body of literature suggests that site-specific mutation rates are dependent on their local sequence context, with sequence-motif associated mutation hotspots identified. Examples of such motifs include CpG dinucleotides (Cuddapah *et al.* 2014; Yang *et al.* 2016) which are correlated with mutation hotspots in mammalian genomes, and repetitive sequences such as homonucleotide runs that are involved in certain mutational events. The mechanism by which such motifs mediate mutation is not well understood, however, and may involve a particular functional role through their location (*e.g.*, splice site) or binding pattern (*e.g.*, known binding of regulatory molecules). Crucially, such motifs may offer a target for therapeutics. Indeed, significant progress has been made in cancer genetics for compounds that target upstream promotor (Weber *et al.* 2005) and transcription factor binding motifs (Wei *et al.* 2006).

With this in mind, we were interested in examining the genomic context of *STXBP1* mutations to elucidate whether their location was characterized by particular recurring motifs. *STXBP1* is one of the most frequent genes that impacts epilepsy related cases and most of the reported pathogenic mutations are *de novo* in origin which in turns provides a unique opportunity for us to investigate the genomic context of such mutations.

## Materials And Methods

### Mutation set collection

**Figure 1:**
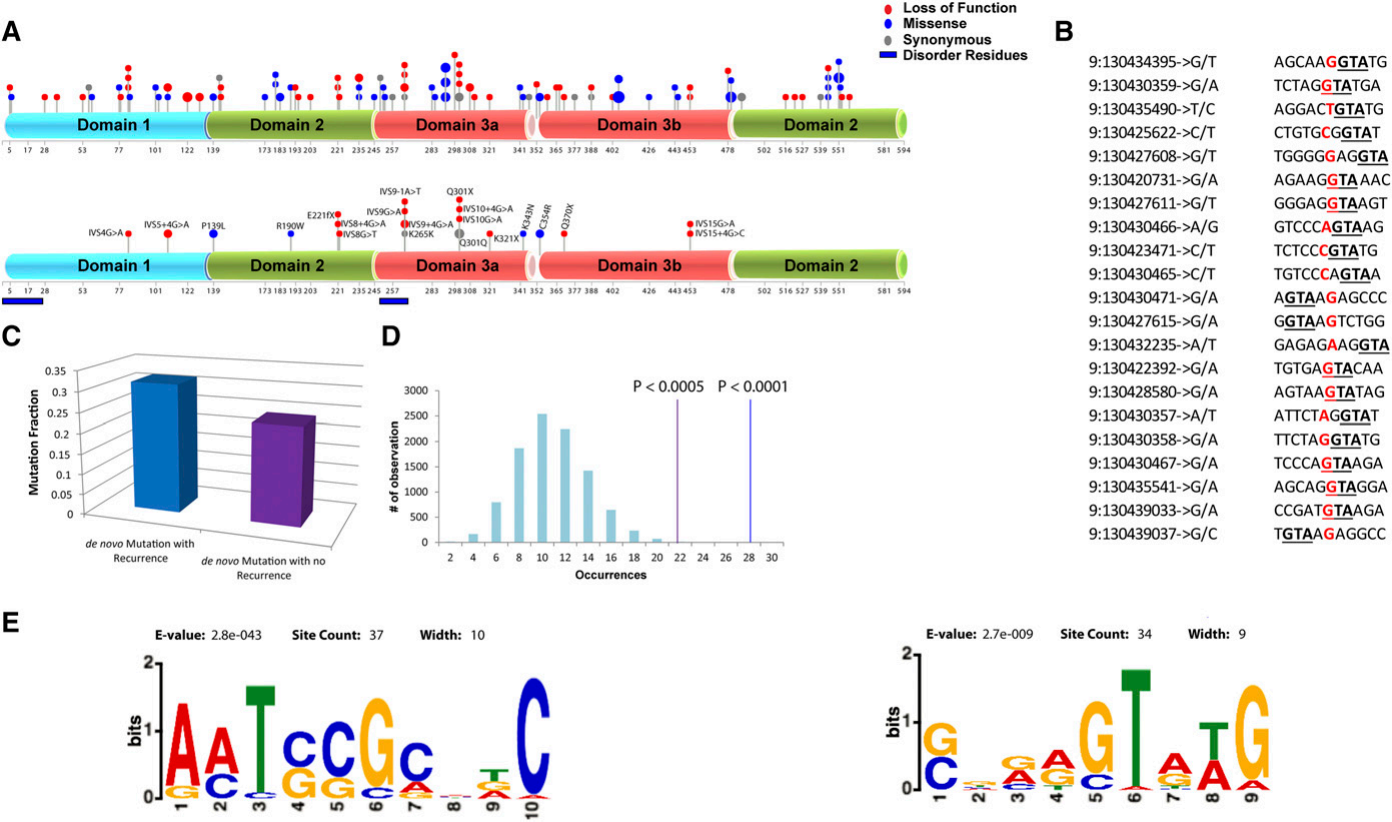
Genomic context analysis if the *STXBP1* gene: (A) Distribution of 136 unique mutations (top) and those mutations associated with the identified ‘GTA’ motif (bottom). The dot size represents the number of recurrent mutations for that specific position. Different domains of the protein and the mutation types are color coded; dark blue bars represent the disordered regions of protein domains; numbers below each domain represents positions of the amino acids. (B) the 21 unique (removing the recurrent) mutations (colored red) associated with the ‘GTA’ motif; (C) shows the fraction of mutations associated with ‘GTA’ motif where the light blue bar represents the fraction with recurrent mutations and dark blue without; (D) results of permutation (Y-axis represents 10,000 randomization) analysis assessing the significance of motifs within the *STXBP1* gene context; the random distribution of motif occurrences is shown in blue bars and the significance of the observed occurrence of motifs within the GTA motif associated mutations (without (red vertical line) and with (green vertical line) recurrent mutations); (E) MEME motif analysis result for all 136 mutations. The diagram shows significant motifs (primarily for “TCC” and “GTA”) identified within the 5bp upstream and 5bp downstream of all reported mutation of *STXBP1* gene.

We have conducted an extensive literature search to identify all *STXBP1* mutations. The literature has described 162 patients (136 SNVs/indels and 26 CNVs) (Uddin *et al.* 2017) with rare (not present in 1000genomes and ExAC frequency <0.0001) or *de novo* heterozygous mutations of *STXBP1* where 140 variants were reported to be *de novo*, comprising variants spread across all three domains of the gene (Figure 1A). In total, 121 unique single nucleotide variants (SNVs) and 15 unique indels have been described. Bearing in mind the clinical importance of this gene, *STXBP1* represents an important gene for the elucidation of its mutational mechanisms.

### Motif occurrence and randomized test

To identify motifs that might be part of a template for molecule binding (*i.e.*, transcription binding factor, enzyme etc), we have conducted frequency estimation of fixed length (*l* = 3) DNA motifs. For each 3-neucleotide length motif, we have estimated the occurrence within an 11 base pair window (5 base pair up and downstream) for a mutation. The co-ordinates of the 136 unique mutations (SNVs and Indels) were used to extract from the human reference genome (build GRCh37, hg19) a 5-base pair window upstream and downstream of each mutation using procedures implemented in BiomaRt. We then undertook a randomized test by computing the occurrences of all possible 3-nucleotide motifs within 5 base pairs (bp) upstream and downstream of each of the independently described *STXBP1* mutations along with 104 rare *STXBP1* coding variants from ExAC (none loss-of-function) (Karczewski *et al.* 2017). This database comprises exome data on 60,706 individuals. A frequency distribution of 10,000 such iterations was generated, and a p-value computed by counting the number of draws greater than or equal to the actual frequency of particular motifs, with significance set at 0.05.

We also conducted an independent genome context analysis using the Multiple EM for Motif Elicitation (MEME) Suite (Bailey *et al.* 2009). This approach uses an expectation-maximization (EM) algorithm that looks for the most significant patterns, described as those that occur most frequently across individual sequences and that have a high rate of similarity. MEME (Bailey *et al.* 2009) reports an E-value, which represents the number of motifs that would be expected by chance if letters in the input sequences were shuffled. As such small E-values are very unlikely to be random.

### Visualization

We have used lollipops software (Jay and Brouwer 2016) to map mutations within the protein domains. Images were collated using adobe illustrator.

### Data availability

The authors state that all data necessary for confirming the conclusions presented in the article are represented fully within the article.

## Results And Discussion

The randomized test implicated significant genomic hotspots with clustering of *de novo*/rare mutations around three motifs ‘ACT’ (*P* < 0.0001), ‘GTA’ (*P* < 0.0001) and ‘TCC’ (*P* < 0.0002) (Figure 1B, depicting the genomic context of the 21 unique mutations with ‘GTA’ motifs; Figure 1C and Table S1). Notably, rare variants (+/−5bp) in ExAC are depleted for these motifs. The significance of the ‘TCC’ and ‘GTA’ associated motifs around the *STXBP1* rare/*de novo* mutations was confirmed using MEME (Figure 1E).

Next, to reduce potential bias introduced by the recurrent nature of some of the mutations, we excluded these recurrent mutations and re-analyzed the 92 unique mutations (SNVs and indels). This re-analysis confirmed the significance (after multiple corrections) of the ‘GTA’ motif (*P* < 0.0005) (Figure 1C). Strikingly, 26.92% of all reported *de novo*/rare SNV mutations in *STXBP1* have a ‘GTA’ motif within 5bp upstream or downstream, implicating a genomic context dependent mutagenesis. We observed that 51.85% (14 out of 27) of the ‘GTA’ mutations are splicing compared to 14.70% (20 out of 136) for mutations overall, with this difference reaching statistical significance (Fisher’s Exact Test (FET) *P* < 0.01, OR = 3.0); we also noted that 11 of these 14 ‘GTA’ associated mutations are *de novo* in origin. Although for all the *STXBP1* mutations there was no enrichment observed for any of the protein domain, the mapping of these ‘GTA’ associated LOF mutations revealed the co-localization primarily within domain 3A (Figure 1A) impacting the disorder region of *STXBP1* protein. There was also no evidence for any difference in type of epilepsy when comparing ‘GTA’ and ‘non-GTA’ mutations. We have analyzed the gender ratio for the 21 unique mutations (27 including recurrent). Gender information was available for 19 cases (out of 27) and we found 73.68% (14 cases) are female and 26.31% (5 cases) are male (Table S1). This female bias was also observed in the original meta-analysis study of 162 cases (Uddin *et al.* 2017).

Our finding of a ‘GTA’ motif in *STXBP1* that is significantly associated with splicing mutations in this gene highlights the importance of genomic context analysis to characterize mutations in neuropsychiatric disease. Our finding shows an identifiable genomic context dependent mutational mechanism for a neuropsychiatric gene, and the robustness is supported by the identification of the same motif using two independent approaches. Previous studies did not find any apparent domain specific or genomic locus clustering of *STXBP1* mutations. However, our genomic context analysis within a +/− 5bp window, demonstrated a motif clustering with splicing mutational events.

Given the ‘rare variant’ architecture of many neuropsychiatric disorders, with many different rare variants spread across individual genes - with each variant conferring susceptibility to the disorder, a context analysis such as ours may provide some clues as to the likely underlying mutational mechanisms and allow more precise phenotype correlation. Importantly, while mutations may at first appear randomly spaced along a particular gene, motif analysis may indicate otherwise. With the availability of high resolution clinical microarray (Uddin *et al.* 2016) and whole genome sequence data (Yuen *et al.* 2017), future studies may unravel unidentified mutational mechanisms by incorporating motif based analyses.

Although DNA motifs have a significant role in gene regulations, it is a major challenge to find the exact mechanism. The role of ‘GTA’ motif into the mechanism of splicing mutation requires further investigation. It is anticipated that these motifs may correspond to a protein binding site, mediating transcriptional regulation, but further molecular studies—for example, engineering motif insertion and deletion—would be required to examine this hypothesis. Moreover, further studies would benefit from examining smaller and larger windows as well as searching for motifs of different sizes (for example 2, 4, and 5 bp motifs). This, however, does carry implication for computational burden, which is one reasons we limited our analysis for 3bp motifs. This notwithstanding, our study has shown that investigating patterns of mutation, and specifically their genomic context, offers the opportunity to begin the scientific process of examining mutational mechanisms, ultimately offering new targets for therapeutic interventions.

## Supplementary Material

Supplemental Material is available online at www.g3journal.org/lookup/suppl/doi:10.1534/g3.118.200080/-/DC1
